# The Emerging Role of PRMT6 in Cancer

**DOI:** 10.3389/fonc.2022.841381

**Published:** 2022-03-04

**Authors:** Zhixian Chen, Jianfeng Gan, Zhi Wei, Mo Zhang, Yan Du, Congjian Xu, Hongbo Zhao

**Affiliations:** ^1^Shanghai Key Laboratory of Female Reproductive Endocrine Related Diseases, Obstetrics and Gynecology Hospital, Fudan University, Shanghai, China; ^2^Department of Obstetrics and Gynecology of Shanghai Medical School, Fudan University, Shanghai, China

**Keywords:** PRMT6, arginine methylation, cancers, regulation, PRMT6 inhibitors

## Abstract

Protein arginine methyltransferase 6 (PRMT6) is a type I PRMT that is involved in epigenetic regulation of gene expression through methylating histone or non-histone proteins, and other processes such as alternative splicing, DNA repair, cell proliferation and senescence, and cell signaling. In addition, PRMT6 also plays different roles in various cancers *via* influencing cell growth, migration, invasion, apoptosis, and drug resistant, which make PRMT6 an anti-tumor therapeutic target for a variety of cancers. As a result, many PRMT6 inhibitors are being utilized to explore their efficacy as potential drugs for various cancers. In this review, we summarize the current knowledge on the function and structure of PRMT6. At the same time, we highlight the role of PRMT6 in different cancers, including the differentiation of its promotive or inhibitory effects and the underlying mechanisms. Apart from the above, current research progress and the potential mechanisms of PRMT6 behind them were also summarized.

## Introduction

Epigenetic regulation includes modification of DNA or histones to affect gene expression and function through methylation, acetylation, phosphorylation, ubiquitination, and chromatin remodeling, while restoring complete DNA sequences. Therefore, epigenetic disorders have become a common mechanism in the occurrence and development of cancer (1). Arginine methylation was first reported in 1971 (2) and was viewed as a key epigenetic regulation of post-translational modification by adding methyl groups to nitrogen atoms of arginine residues in polypeptides, and participating in the regulation of various cellular processes, including splicing, transcription, translation, and signaling (3, 4). It changes the metabolic landscape of cells and further leads to cancer metastasis (5), DNA damage (6), and parasitic infection (7). But interests in the post-translational modification (PTM) did not expand until PRMT1 was cloned in the mid-1990s. Compared with other PTMs, the research progress of arginine methylation is relatively slow mainly due to the lack of reliable arginine-methyl antibodies and effective small-molecule inhibitors. In eukaryotes, there are three distinct forms of arginine methylation, monomethylarginines (MMAs), asymmetric dimethylarginines (ADMAs) and symmetric dimethylarginines (SDMAs). The production of larger and more hydrophobic residues during methylation may influence the interactions with other proteins or nucleic acids (8–10).

A large number of recent reports have confirmed that protein arginine methyltransferases (PRMTs) are a family of enzymes that methylate arginine residues of substrate proteins, and the main mechanism of PRMTs affecting cell activity is epigenetic regulation. Much of this activity can be attributed to their ability to methylate histone tails. However, at the same time, the PRMT family can also methylate non-histone proteins in transcription (11, 12). To sum up, the role of PRMT family can be reflected in many aspects, such as the major regulators of epigenetic-mediated gene expression, mRNA splicing, DNA damage responses, stem cell function, and immune responses (11, 13). PRMTs can be subdivided according to their methylation pattern: type I PRMTs (PRMT1, 2, 3, 4, 6 and 8) mainly catalyze the synthesis of MMA and ADMA; type II PRMTs (PRMT5 and PRMT9) catalyze the synthesis of MMA and SDMA; and type III PRMT (PRMT7) regulates monomethylation. There is another type IV PRMT that produces δ-NG-monomethylarginine in fungi (14, 15). PRMTs are recruited to target genes by transcription factors and act as a part of multicomponent transcription complexes. Although cancer-related mutations in PRMTs are uncommon, PRMTs expression levels are usually elevated in patients and are associated with poor prognosis (16, 17). These findings have led to a widespread interest in PRMTs as new cancer drug targets.

Protein arginine methyltransferase 6 (PRMT6) is a type I PRMT that asymmetrically dimethylates protein substrates on arginine residues and has functions in epigenetic regulation of gene expression (18–21), alternative splicing (13, 22), development and differentiation (23–25), DNA repair (26), cell proliferation and senescence (27–29), DNA methylation (30), mitosis (31, 32), inflammation (33–35), congenital antiviral immunity (36), spermatogenesis (37), transactivation of nuclear receptors (13, 38) and cell signaling (39, 40). In addition, the dysregulation of PRMT6 is also associated with viral diseases (41, 42), cancer (43) and cardiac dystrophy (44). At the same time, PRMT6 plays a role in a variety of hitherto unidentified cellular functions (45). PRMT6 is predominantly located in the nucleus, in sharp contrast to PRMT3 and PRMT5, which are predominantly present in the cytoplasm. Other members of the PRMT family are present in both the nucleus and cytosol. PRMT6 is expressed in a variety of tissues, and significantly increased in kidney and testes (21). Moreover, it also exists in regulatory DNA regions of important cell cycle regulators such as CDKN1A, CDKN1B, CDKN2A and p53, thus acts as a transcription inhibitor in these regions (28, 29, 46, 47). It produces asymmetric dimethylation in histone 3 at arginine 2, arginine 17, arginine 42 (H3R2me2a, H3R17me2a, and H3R42me2a) (18–20, 48, 49), arginine 26 (H2AR26me2a) (50) and is involved in epigenetic regulation of gene expression. In addition, PRMT6 can methylate a variety of non-histone proteins and regulate a variety of biological functions.

**Figure 1:**
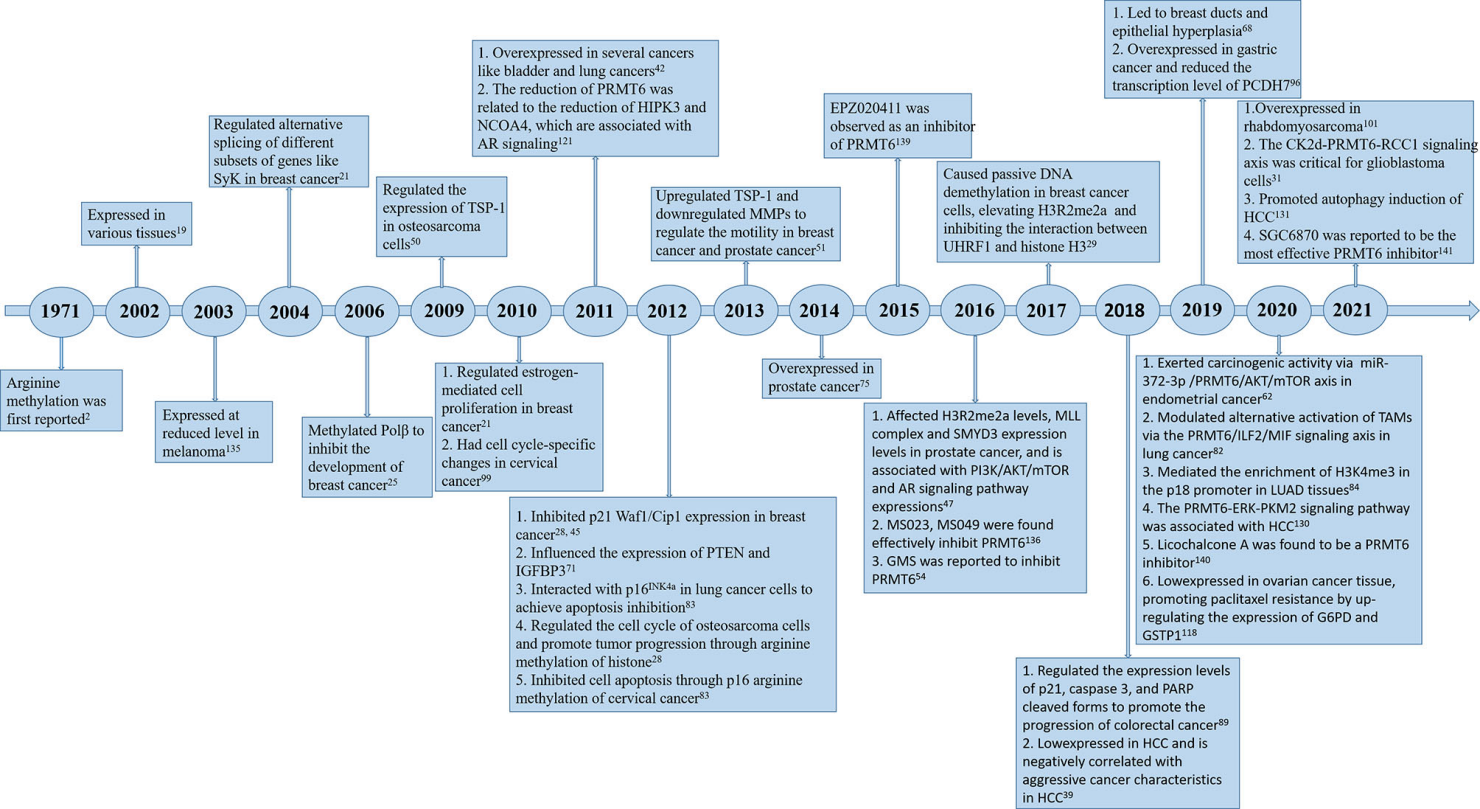
Timeline of *PRMT6* research. A brief history of functional and pharmacological studies of PRMT6.

Since PRMT6 was identified more than 20 years ago, many studies have been performed to identify the characteristics and molecular functions of PRMT6 in cancer (**Figure 1**). Asymmetric dimethylation of histone H3 at arginine 2 (H3R2me2a) was catalyzed by protein arginine methyltransferase 6 (PRMT6). PRMT6-dependent H3R2me2a can be detected on active genes at promoters and enhancer sites. That is, PRMT6 interferes with the deposition of adjacent histone markers through H3R2me2a and regulates the activity of important differentiation-related genes through reverse transcriptional effects. Depending on its genomic location, H3R2me2a can exhibit either inhibitory or activating properties: transcriptional inhibition at promoters and transcriptional activation at enhancers, respectively. Therefore, PRMT6 can promote or inhibit cancer development, and its effect is not single and fixed (51). For example, PRMT6 knockdown can significantly inhibit the growth of bladder cancer and lung cancer cells (43). In addition, PRMT6 knockdown results in upregulation of tumor suppressor genes p21 and p27 (28, 29, 46). Therefore, PRMT6 promotes cell growth and prevents senescence, thus becoming an anti-tumor therapeutic target for various types of cancer. Furthermore, PRMT6 is involved in regulating gene expression of TSP-1, a potent natural inhibitor of angiogenesis (52, 53), RUNX1, a group of target genes involved in hematopoietic stem/progenitor cell differentiation (54), and genes participated in maintaining embryonic stem cell characteristics (24). In addition, studies have confirmed that PRMT6 is involved in regulating drug resistance of tumor cells. p21^CDKN1A^, as an effective inhibitor of cyclin-dependent kinase (CDK), regulates the cell cycle in a p53-dependent manner in response to a variety of stimuli, including DNA damage. PRMT6 methylates p21 at arginine 156 and promotes phosphorylation of threonine 145 on p21, resulting in the increased cytoplasmic localization of p21. The cytoplasmic presence of p21 makes cancer cells more resistant to cytotoxic drugs (55). PRMT6 can also act as a limiting factor of viral replication, acting on the pathogenesis of human immunodeficiency virus through methylation of TAT and other HIV proteins (41). In this review, we will summarize some recent studies on PRMT family, and focus on the different roles of PRMT6 in tumor. We will also note that PRMT6 is a potential target for cancer therapy.

## Tumor Promoting Roles of PRMT6 in Cancers

### Endometrial Cancer

Endometrial carcinoma (EMC) ranks the fourth most frequently diagnosed cancer among women. In 2020, 130,051 newly diagnosed cases and 29,963 EMC-related deaths were estimated (56). *In vitro* and *in vivo* studies showed that PRMT6 was up-regulated at mRNA and protein levels in EMC. PRMT6 exerts carcinogenic activity by activating the AKT/mTOR pathway, promoting cell proliferation and migration in EMC. Data showed that the expression of PRMT6 in EMC was controlled by miR-372-3p, which targeted the 3’UTR of PRMT6 promoter to inhibit its expression. Down-regulation of miR-372-3p and its inhibitory effect on proliferation and migration of endometrial cancer cells have been reported (57). MiR-372-3p functions through the AKT/mTOR pathway, studies have shown that PHLPP2 was the theoretical target gene of miR-372. PHLPP2 belongs to a novel Ser/Thr protein phosphatase family that negatively regulates AKT, PKC, MAPK, and Mst1-activated signaling pathways and plays a central role in maintaining cell survival inhibition. This was confirmed by the fact that miR-372 gene knockout inhibited the phosphorylation levels of major components of the PI3k/AKT pathway, including AKT, mTOR, and P70S6K (58). Therefore, it can be inferred that miR-372-3p/PRMT6/AKT/mTOR axis can serve as both a prognostic factor and therapeutic target for EMC (59).

### Breast Cancer

Breast cancer is one of the most widespread invasive cancers, accounting for 25-30% of new cancers in women, ~15% of cancer-related deaths in women, and ~6.5% of all cancer-related deaths (60). In previous studies on the relationship between PRMTs family and breast cancer, PRMT1 is verified overexpressed in breast cancer tumor samples, and its expression degree is related to tumor grade (43, 61). Meanwhile, CARM1 was found to methylate a large number of proteins with a variety of biological functions, including regulation of intracellular estrogen receptor-mediated signaling, chromatin organization and chromatin remodeling. CARM1 can recruit the coactivator protein tumor-domain-containing protein 3 (TDRD3) to its binding enhancer through hypermethylation of these proteins to activate the estrogen/ERα-target genes. Therefore, CARM1 can promote the proliferation of ERα -positive breast cancer cells and tumor growth in mice *in vivo* (62). PRMT1 is also overexpressed in breast cancer tumor samples, and its expression degree is related to tumor grade (43, 61).

Since the role of CARM1 in estrogen-dependent breast cancer was confirmed (62), PRMT6-siRNA-1 assay showed that PRMT6 also played a role in estrogen signaling, and the survival rate of MCF-7 cells was significantly reduced, participating in estrogen-stimulated ERα-expressing breast cancer cell proliferation (13). Since PRMT6 and CARM1 can play a synergistic role in estrogen signal transduction, when they are knocked out together, a synergistic effect is observed in the regulation of estrogen-induced proliferation (13). PRMT6 has also been shown to regulate alternative splicing of different subsets of genes in MCF-7 cells in hormone-independent way. For example, in VEGF genes, whilst reduction of PRMT6 transcription increases the ratio of VEGF189 to VEGF165 by more than 2 times. In Syk genes, PRMT6 knockout will lead to a significant increase in the ratio of Syk [L]: Syk [S] (22). Therefore, it can be concluded that PRMT6 is an essential component of estrogen signaling pathway in breast cancer cells. In addition to regulating transcription initiation in estrogen signaling pathway, it can also affect various aspects of RNA progression, especially alternative splicing (13). Meanwhile, PRMT6 negatively regulates DNA methylation, and the upregulation of PRMT6 contributes to global DNA hypomethylation in cancer. Depletion or inhibition of PRMT6 could restore global DNA methylation of MCF7 cells. Mechanistically, PRMT6 overexpression impairs chromatin binding of UHRF1, a cofactor of DNMT1, leading to passive DNA demethylation. This effect may be due to elevated H3R2me2a, which inhibits the interaction between UHRF1 and histone H3 (30). PRMT6 can also directly inhibit the p21 promotor. PRMT6 gene knock-down (KD) results in a p21 derepression in breast cancer cells, which is p53-independent, and leads to cell cycle arrest, cellular senescence and reduced growth in soft agar assays, and severe combined immune deficiency (SCID). Bypassing the p21-mediated arrest rescues PRMT6 KD cells from senescence and restores their ability to grow on soft agar. And it directly inhibits p21 Waf1/Cip1 expression by targeting the promoter gene region (29, 46). It has also been found that K5-driven conditional overexpression of PRMT1, CARM1 and PRMT6 leads to breast ducts and epithelial hyperplasia to varying degrees at different time points. In the context of Neu-induced carcinogenesis, overexpression of PRMT1 and PRMT6 significantly accelerated breast tumor onset, while CARM1 increased tumor progression only at tumorigenesis. These results suggest that all three type I PRMTs have carcinogenic activity that predisposed mouse mammary gland to tumorigenesis, and support the targeting of these PRMTs for breast cancer patients (63). PRMT6 methylation of H3R2 promotes transcriptional inhibition of HoxA10 (52), a protein whose upregulation promotes increased p53 expression and reduced invasive potential in breast cancer (64).

In addition, Affymetrixexon microarray (Santa Clara, CA) was used to demonstrate that PRMT6-dependent gene signature influences long-term survival in breast cancer patients, which included (i) reduced level of the tumor inhibition, PTEN in breast cancer patient samples and increased PTEN mRNA expression after loss of PRMT6 in breast cancer cells, and (ii) differential splicing of genes involved in centrosome targeting, cell invasion, apoptosis, p21-interacting proteins, and other genes which involved in cell cycle regulation. In addition, they demonstrated that abnormal expression of PRMT6 and PRMT6-dependent gene signature is associated with poorer clinical prognosis in patients with ER+ breast cancer (22). Experiments have also observed that PRMT6 mRNA expression level in the invasive ductal carcinoma (IDC) breast cancer is significantly lower than that in normal breast tissues. When PRMT6 is knocked down in the MCF-7 cell lines, the expressions of PTEN and IGFBP3 are increased. PTEN is a tumor suppressor gene that inhibits the PI3K pathway (65). Loss of PTEN leads to the activation of many kinases and subsequent cell cycle progression (66), and IGFBP3 has an anti-proliferative effect and induces apoptosis of breast cancer cells (67). This suggests that lower PRMT6 expression may lead to increased expressions of PTEN and IGFBP3, decreased cell cycle progression and increased apoptosis of breast cancer cells (22). Therefore, PRMT6 plays an essential role on the development, metastasis, treatment, drug resistance and many other aspects of breast cancer.

### Prostate Cancer

Prostate cancer (PCa) is the fourth most frequently diagnosed malignant tumor in men and the second leading cause of cancer-related mortality worldwide which just behind lung cancer (56). Because intracellular CARM1 levels are important for estrogen and androgen receptor signaling, it has previously been shown to be altered in breast and prostate cancer tissues (68, 69). PRMT6 was also found to be overexpressed in prostate tumor tissue, which was distinguishable from normal prostate tissue (70). Overexpression at the transcription and protein levels was associated with poorer disease-free survival in PCa, suggesting a carcinogenic effect. Stable PRMT6 knockdown attenuated the malignant phenotype in PC-3. At the molecular level, PRMT6 silencing was associated with decreased H3R2me2a levels and increased expression of the MLL complex and SMYD3. PRMT6 silencing increased p21, p27 and CD44, decreased the expression of MMP-9, which was associated with downregulation of PI3K/AKT/mTOR and increased androgen receptor (AR) signaling pathway (71). The potential clinical relevance of restoring AR expression in Sh-PRMT6 PC-3 suggests that PRMT6 inhibition may re-sensitize androgen-insensitive tumor cells to ADT, providing a new approach for the treatment of castration-resistant prostate cancer (CRPC) (71).

### Lung Cancer

Lung cancer is the leading cause of cancer-related deaths worldwide (18% of the total cancer deaths) with an estimated 1.6 million deaths each year and imposes a heavy burden on health care system (56, 72). Previous studies have proved that PRMT1, PRMT5 and PRMT7 in the PRMT family are all highly expressed in lung cancer tissues (43, 73–76). It was confirmed that PRMT6 is upregulated in lung cancer and can promote the growth of tumor cells (43). Depletion of PRMT6 can reduce cell proliferation, cell migration and anchorage-independent growth of NSCLC cells. There is a protein-protein interaction (PPI) between PRMT6 and interleukin-enhancer binding protein 2 (ILF2), and the PRMT6-ILF2 signaling axis is a novel regulator of macrophage migration inhibitor factor (MIF). Therefore, PRMT6 promotes lung tumor progression by modulating the alternate activation of tumor-associated macrophages (TAMs). Targeting the newly identified PRMT6/ILF2/MIF axis may open new possibilities for lung cancer intervention (77). PRMT6 can also interact with p16, overexpression of PRMT6 can counteract the cell cycle arrest at G1 phase caused by p16 in NSCLC A549 cells and decrease the association intensity of p16-CDK4, suggesting that PRMT6 probably achieves its cell apoptosis restraint role in NSCLC through p16 arginine methylation and provide a new idea for NSCLC treatment (78). Besides, it was also found that PRMT6 gene knockdown can significantly increase the enrichment of H3K4me3 in the p18 promoter in lung adenocarcinoma (LUAD) tissues, which leads to upregulation of p18, thus mediating G1/S phase cell cycle arrest and inhibiting the proliferation of LUAD cells *in vitro* and *in vivo*. This negative correlation between PRMT6 and p18 suggests that p18 may be a downstream target of PRMT6, which acts as an oncogene in the disease and epigenetically inhibits the expression of p18 and interferes with G1/S phase transition of LUAD cells (79). In terms of treatment, a study has identified the combination of PARP inhibitors and type I PRMT inhibitors offers new therapeutic opportunities for MTAP-negative NSCLC and cancers that are resistant to PARP inhibitors (80).

### Colorectal Cancer

Colorectal cancer (CRC) is one of the most commonly diagnosed cancers in the world and a leading cause of cancer death, among men, colorectal cancer ranks third in both incidence and mortality (56). Previously, overexpression of three major types of type I PRMTs has been observed in CRC, including PRMT1 (81) and CARM1 (82, 83). PRMT6 is observed up-regulated in CRC tissues, its activity plays a key role in tumor cell differentiation. PRMT6 gene knockdown (KD) promotes CRC cells apoptosis by upregulating the tumor suppressor p21 protein in CRC cells. It also increases the expression of the cleavage forms of caspase 3 and PARP, thereby inhibiting the growth and colony formation of CRC cell lines, which indicated that PRMT6 played an important role in promoting the proliferation and progression of CRC (84). Recently, the protein arginine methyl transferase (PRMT) type 1 inhibitor MS023 was found to be an effective inducer of alkaline phosphatase (ALP) activity promoting cell differentiation phenotype, significantly delaying the growth of CRC cells. Therefore, it was selected as a probe with the potential to modulate the CRC phenotype. PRMT1 has been confirmed as a target of MS023, and PRMT6, as a member of type 1 PRMT family, is also promising as a new anticancer target (85).

### Osteosarcomas

Osteosarcoma is the most common malignant primary bone tumor in children and adolescents, characterized by the formation of immature bone or osteoid tissue from the sarcoma cells that develop mainly in the long bones (86, 87). It has been confirmed that overexpression of CARM1 in the PRMT family promotes human osteosarcoma cell proliferation through the PGSK3β/β -catenin/cyclin D1 signaling pathway (88). In PRMT6-deficient osteosarcoma cells U2OS cells, the expression of TSP-1 gene promoter was increased and the loss of H3R2me2a and the corresponding increase of H3K4me3 were observed at the TSP-1 promoter, inhibiting the migration of U2OS cells. These results suggest that PRMT6 regulates the expression of TSP-1 in osteosarcoma cells by regulating epigenetic markers at the TSP-1 promoter level (52). It has been found that down-regulation of PRMT6 leads to up-regulation of p21 and p27, two members of the cyclin-dependent kinase (CDK) inhibitor CIP/KIP family, and accumulation of human osteosarcoma cell line U2OS at the G2 checkpoint. PRMT6 also involves the methylation of arginine-2 of histone H3, so PRMT6 can regulate the cell cycle of osteosarcoma cells and promote tumor progression through arginine methylation of histone (29).

### Bladder Cancer

As one of the most common malignant tumors of the urinary system, bladder cancer (BCa) ranks tenth with high morbidity and mortality worldwide, with approximately 573,000 new cases and 213,000 deaths (56). It was found that PRMT1 and PRMT6 were significantly upregulated in bladder tumor tissues. When PRMT1 and PRMT6 genes were knocked down, the growth of bladder cancer cell lines (SW780 and RT4) was significantly inhibited, and the cells in the S phase were significantly reduced, while those in G0 and G1 phases were increased simultaneously. These results indicated that PRMT1 and PRMT6 play an important role in the G1-S transformation of bladder cancer cells. Meanwhile, real-time quantitative RT-PCR analysis of gene microarray data suggested that PRMT1 and PRMT6 could lead to the carcinogenesis of bladder cancer by regulating RNA processing and DNA replication. Therefore, PRMT1 and PRMT6 may be a promising target for bladder cancer therapy, and their inhibitors may be ideal candidates for molecular targeted therapy of bladder cancer (43).

### Gastric Cancer

Gastric cancer (GC) is the fifth most commonly diagnosed cancer type and the third leading cause of cancer-related deaths, with more than 1 million new cases diagnosed and more than 780,000 deaths per year (8.2% of all cancer deaths) (56). In the studies on the effect of PRMT family on GC, it was previously concluded that PRMT5 (89) and PRMT8 (90) expressions were both significantly increased in GC tissues, and were both significantly correlated with the short-term survival rate of GC patients. It was also found that the expression level of PRMT6 in GC was significantly higher than that in non-cancer tissues, and overexpression of PRMT6 enhanced the aggressiveness of gastric cancer cells and increased the expression level of H3R2me2a in GC cells through the direct pathway of transcriptional inhibition of tumor suppressor gene procadherin 7 (PCDH7), and the expression level of H3R2me2a was an independent prognostic indicator of GC. Therefore, PRMT6 may have carcinogenic properties, and its overexpression may contribute to GC progression and become a new therapeutic target for GC (91).

### Cervical Cancer

Cervical cancer (CC) is a serious and common gynecological malignant tumor disease located in the cervix, with high morbidity and mortality. In recent years, the age of onset is gradually younger, and the incidence is on the rise (92). Previous studies have shown that PRMT5, a type II PRMT, is highly expressed in cervical cancer, and arginine methyltransferase inhibitor 1 (AMI-1) can inhibit solid tumors of cervical cancer by targeting PRMT5 (93). At the same time, PRMT8 expression was also observed to be elevated in cervical cancer (90). It was found that the level of PRMT6 was upregulated from G0/G1 to G1 phases in HeLa cells and recovered to the level of G1 phase after the M phase. Meanwhile, aDMA-methylated 64kDA CstF-64, which is involved in mRNA metabolism, 80kDa hnRNPR and their 68kDa subfamily, which are involved in post-transcriptional processing of mRNA, and 25 kDa TPI regulating glycolysis and gluconiogenesis were also upregulated in G0/G1 phase, suggesting that PRMT6 has cell cycle-specific changes in cervical cancer cells and is most likely to modulate the cellular growth and proliferation during HeLa cell cycle (94). In HeLa cells, PRMT6 can also interact with p16 to methylate it and reduce the association of p16 and CDK4, suggesting that PRMT6 inhibits cell apoptosis through p16 arginine methylation, which also makes p16-associated gene therapy for cervical cancer a possible new strategy (78).

### Rhabdomyosarcoma

Rhabdomyosarcoma (RMS) is a rare malignancy neoplasm that originates in the mesenchyme or neuroectoderm, which is arisen from skeletal muscle progenitor cells. Despite its low incidence (4.5 cases per million children), it is the most common soft tissue sarcoma in children, nearly 20% of patients presenting with locally aggressive and/or metastatic disease (95). The expression of PRMT1, CARM1, PRMT5, PRMT7, PRMT8 and PRMT9 were all elevated in rhabdomyosarcoma (96), CARM1 and its direct interaction with histone acetyltransferase PCAF jointly were also detected to exert an increased expression of myogenin gene and lead to rhabdomyosarcoma cell differentiation (9). It was observed that the expression of gene encoding PRMT6 was increased in rhabdomyosarcoma cell lines, and PRMT inhibitors (AMI-1 and SAH) effectively reduced the invasive phenotype of RMS cells by inhibiting the proliferation rate, cell viability and colony formation ability of rhabdomyosarcoma cell lines. These inhibitors also attenuate the activity of the PI3K-Akt signaling pathway, resulting in decreased levels of cyclin D1 and Bcl-xL and increased level of GADD45G protein, thus halting the cell cycle and promoting apoptosis (96). Therefore, PRMT6 is expected to be a new therapeutic target for rhabdomyosarcoma.

### Others

The tumor-promoting effect of PRMT6 is also seen in other systems, such as the blood system, and can be seen in leukemia. Leukaemias are commonly divided into chronic or acute leukaemias and as lymphocytic or myelogenous leukaemias; the subtypes include chronic lymphocytic leukemia (CLL), chronic myeloid leukemia (CML), acute myeloid leukemia (AML), and acute lymphocytic leukemia (ALL) among others (97). More and more evidences show that PRMT family plays an important role in malignant hematopoiesis. PRMT1 has been found to interact with AML1-ETO, which acts as an oncogenic transcription factor and occurs in 15% of *de novo* AML cases, and methylate its fusion protein, thereby promoting transcriptional activation and self-renewal capability (98). PRMT1 can also collaborate with some MLL fusion proteins in MLL leukemia, and the enzyme activity of PRMT1 has been shown to be critical for MLL-mediated transformation (99–101). It has also been found that increased activity of PRMT5 promoted the growth of AML *in vitro* and *in vivo*, while downregulation of PRMT5 decreased the growth of AML (102), and elevated levels of PRMT5 in some leukemia and lymphoma cells lead to H3R8 and H4R3 hypermethylation and transcriptional silencing in promoter regions of the RB tumor suppressor family, which suggest that PRMTs can regulate the expression of miRNAs (103). It was also found that N1-(2-((2-chlorophenyl)thio)benzyl) -N1-methylethane-1,2-diamine (28d, DCPR049_12), a highly potent inhibitor of type I PRMTs, effectively inhibited cell proliferation and reduced asymmetric arginine dimethylation levels in several leukemia cell lines. 28d, as a potent inhibitor, demonstrates the cell killing mechanisms in both cell cycle arrest and apoptotic effects as well as downregulation of the pivotal mixed lineage leukemia (MLL) fusion target genes such as HOXA9 and MEIS1, reflecting the critical roles of type I PRMTs in MLL leukemia (104). It was also found that the transcriptional co-repressors PRMT5 and PRMT6 were overexpressed in hematocarcinoma and inhibited the expression level of tumor suppressor genes (5).

This effect has also been seen in brain tumors such as glioblastoma. Gliomas are the most common malignant tumors of the central nervous system with high intra- and inter-tumor heterogeneity, and the most destructive form of glioma is grade IV astrocytoma, known as glioblastoma (GBM) (105). It has been found that abnormal expression of PRMT is associated with the development of brain tumors such as glioblastoma and medulloblastoma, for example, PRMT1 (106) is known as a contributor to the development of GBM, PRMT2 is also speculated to be involved in the pathogenesis of GBM by promoting cell stemness (107), CARM1/PRMT4 can also regulate the production of miR-17-92a, thus affecting the differentiation and production of neuronal and glial cells (108), PRMT8 depletion can increase cellular markers associated with gliomagenesis (109), the increased expression of PRMT5 has also been implicated in tumorigenesis and is associated with worse GBM prognosis (110). PRMT6 and subunits of polycomb repressor complexes 1 and 2 bind regulatory regions of HOXA genes were found to affect neuronal differentiation (111). PRMT6 was also found to promote RCC1 chromatin association, thereby enhancing the mitotic activity of GBM cells, while casein kinase 2α (CK2α) phosphorylates and stabilizes PRMT6. The CK2α-PRMT6-RCC1 signaling axis is critical for GBM cell mitosis. Inhibition of PRMT6 can reduce the tumogenesis of GBM cells and enhance the cytotoxic activity of radiotherapy (RT) (32).

## Tumor Suppressive Roles of PRMT6 in Cancers

### Ovarian Cancer

Ovarian cancer is a malignant tumor only found in female reproductive system, with high morbidity and mortality, the most dominant pathological subtype is epithelial OC, including five major tissue types, differ in origin, pathogenesis, molecular changes, risk factors, and prognosis (112).. Previous studies have found that in the PRMT family, the expression level of PRMT8 in ovarian cancer is significantly increased and is associated with increased survival rate of patients (90). Compared with normal ovarian tissues, PRMT6 mRNA expression levels were decreased, while glucose-6-phosphate dehydrogenase (G6PD) and glutathione S-transferase P1 (GSTP1) protein expression levels were significantly up-regulated, and the three changes were correlated with each other. GSTP1 can detoxify several anticancer drugs and mediate specific S-glutathionylation of ER-resident proteins to induce chemotherapy resistance in tumors and stress response, suggesting that the decrease of PRMT6 expression and subsequent increase of G6PD expression give ovarian cancer cells resistance to paclitaxel by regulating GSTP1 expression. These results suggest that PRMT6 may be a new potential target for overcoming paclitaxel resistance in ovarian cancer (113).

### Breast Cancer: Invasive Ductal Carcinoma

Experiments have shown that the reduction of PRMT6 in the MCF-7 and T47D ER+ cell lines leads to the reduction of HIPK3 and NCOA4, two transcriptional co-activators originally associated with androgen receptor (AR) signaling. This suggests that in addition to the co-activation of steroid hormone-dependent genes, PRMT6 is also involved in the expression of genes that promote steroid hormone signaling. Interestingly, AR signaling in ER+ breast cancer is thought to be associated with favorable prognosis (114, 115), and the reduction of HIPK3 and NCOA4 may negatively affect AR signaling and ER+ breast cancer prognosis. Conversely, the reduction of the NCOA4 full-length isoform in MCF-7 cells was associated with increased breast cancer metastasis (116), while NCOA4 knockdown in MCF-7 cells led to reduced cell proliferation (117). Therefore, the exact role NCOA4 plays in breast cancer is not fully established and requires further research (22). PRMT6 was observed overexpressed in breast cancer MCF7 cell lines, while the levels of thrombospondin-1 (TSP-1), an effective natural inhibitor of angiogenesis, were highly up-regulated in PRMT6-overexpressing cells. Significant down-regulations of MMP-2 and MMP-9 were also observed in PRMT6-overexpressing cells. Compared with control GFP expressing cells, the growth rate and colony formation ability of PRMT6 overexpressed cells were significantly reduced, which suggest that PRMT6 overexpression is involved in the regulation of motility and invasion in human breast cancer cells through up-regulation of TSP-1 and down-regulation of MMPs (53).

However, these results are inconsistent with previous observations that TSP-1 is a transcriptional inhibitory target of PRMT6 and blocks secretory TSP-1 erythrocyte migration in PRMT6-deficient osteosarcoma cells (U2OS) (52). One possible reason for this analysis is that the cellular system (MCF7 vs U2OS) is different between the two studies, the other is the difference between PRMT6 overexpression system and PRMT6 knock-down system. In knock-down system, the absence of PRMT6 could not write the inhibitory epigenetic marker H3R2me2a on the TSP-1 promoter region, resulting in increased TSP-1 expression. However, in the overexpression system, overexpressed PRMT6 may inhibit the expression of some inhibitory complexes of TSP-1 transcriptional inhibition by generating H3R2me2a on their expression region, such as txr-1 and id-1 (118), thus transforming into TSP-1 transcriptional activation (53). It was also found that PRMT6 methylates DNA polymerase β (Polβ), an enzyme involved in DNA base excision and repair. PRMT6 methylation of Polβ arginine residues 83 and 152 is required to stimulate Polβ’s DNA-binding ability and promote its processability (26). Low Polβ mRNA and protein expression was associated with breast cancer incidence, higher tumor grade, positive lymph node status, increased ER+ tumor aggressiveness, and poor patient survival (119). This suggests that PRMT6 methylation of Polβ is a mechanism that promotes genomic stability and thus may inhibit the development of breast cancer. Therefore, PRMT6 may be served as a therapeutic marker for breast cancer, but its exact effects on breast cancer need further study (120).

### Hepatocellular Carcinoma

Hepatocellular carcinoma (HCC) is one of the most common cancers worldwide, and the main type of primary liver cancers, accounting for approximately 90% of human liver cancer and the third leading cause of cancer-related deaths (121). PRMT2 (122), PRMT9 (123) had previously been shown to accelerate the development, invasion and metastasis of hepatocellular carcinoma, however, PRMT5 was found to inhibit the growth of HCC (124). It has been found that PRMT6 is frequently down-regulated in HCC, and its expression is negatively correlated with aggressive cancer characteristics in HCC patients. In a DEN+CCL4HCC-induced PRMT6 knockout mouse model, deletion of PRMT6 expression exacerbates the occurrence of liver tumors. The silencing of PRMT6 promotes tumor initiation, metastasis, and anti-therapeutic potential in HCC cell lines and patient-derived organoids. PRMT6 interacts with CRAF on arginine 100 to interfere with its binding to RAS/RAF binding domain, reduce its RAS binding potential and alter its downstream MEK/ERK signaling, thereby maintaining a key inhibitory function of HCC cells (40). The use of 2-deoxyglucose (a glycolysis inhibitor) reverses tumorigenicity and sorafenib resistance mediated by PRMT6 deficiency in HCC. Most tumor cells utilize aerobic glycolysis (the Warburg effect) to support anabolic growth, promoting tumorigenicity and drug resistance. PRMT6 regulates aerobic glycolysis in HCC through nuclear relocalization of pyruvate kinase M2 subtype (PKM2), a key regulator of the Warburg effect. PRMT6 methylates CRAF at arginine 100 and interferes with its RAS/RAF binding potential, thereby altering the ERK-mediated translocation of PKM2 to the nucleus. REST is a novel target of PRMT6 hypoxia, linking PRMT6 with hypoxia to drive glycolysis events. Use of the glycolysis inhibitor 2-deoxyglucose (2DG) reverses tumorigenicity and sorafenib resistance caused by PRMT6 defect mediated glycolysis events in HCC. Therefore, the regulatory axis of PRMT6-ERK-PKM2 has a mechanistic association with tumorigenicity of HCC, sorafenib resistance, and Warburg effect of tumor cells, and is an important determinant factor (125). Since autophagy is a key survival factor for cancer cells, it can maintain cellular homeostasis by degrading damaged organelles and unwanted proteins and support cellular biosynthesis in response to stress.

It was found that deficiency of PRMT6 promotes autophagy induction in HCC in response to nutrient/oxygen starvation and drug-induced stress, the catalytically active domain of PRMT6 plays an important role in autophagy regulation of HCC. The enhanced autophagic flux of HCC cells was negatively correlated with the expression of PRMT6, and the catalytic domain of PRMT6 was critical in mediating these autophagy activities. PRMT6 physically interacts and methylates BAG5 to enhance the degradation of its interaction partner HSC70 (a well-known autophagy participant), and a reverse correlation between PRMT6 and HSC70 expression in HCC tissues was observed. The therapeutic potential of gene targeting BAG5 to reverse tumogenesis and sorafenib resistance mediated by PRMT6 defects in HCC has also been demonstrated *in vivo* models. In conclusion, PRMT6 deficiency regulates BAG5-related HSC70 stability through post-translational methylation of BAG5, thereby inducing autophagy to promote tumorigenicity and cell survival in the malignant microenvironment of HCC tumors. Therefore, targeting BAG5 by inhibiting autophagy and inducing the sensitivity of HCC cells to sorafenib may be an attractive strategy for the treatment of HCC (126). Therefore, PRMT6 can be viewed as a new target for liver cancer research, and its specific mechanism of action remains to be further studied.

### Prostate Cancer

It was observed that the growth rate and colony formation ability of PRMT6 overexpressed cells were significantly reduced in PC3 cell lines, and the expression of thrombospondin-1 (TSP-1), an effective natural inhibitor of angiogenesis, was highly up-regulated in PRMT6-overexpressing cells. When TSP-1 was specifically knocked down, the inhibition of migration and invasion by overexpression of PRMT6 was significantly saved. Concomitantly, down-regulation of MMP-2 and MMP-9 expressions were observed in PRMT6-overexpressing cells. These results indicated that PRMT6 overexpression could inhibit the migration and invasion of prostate cancer cells by up-regulating TSP-1 and down-regulating MMPs (53).

### Melanoma

Malignant melanoma (MM) is a skin tumor that originates in melanocytes and is responsible for melanin production and its metastasis to keratinocytes (127). Previous study has found that the expression of most PRMTs in primary tumours and metastases remains unchanged compared with normal melanocytes, and only PRMT4/CARM1 was significantly induced during tumor development (128). PRMT1 has also been found to be overexpressed in human melanoma, and PRMT1 may regulate tumor growth and metastasis by targeting activated leukocyte cell adhesion molecule (ALCAM) (129). PRMT6 was found to be expressed at reduced levels in melanoma compared to melanocytes, early studies have shown that the expression of methylthioadenosine phosphorylase (MTAP), which is involved in tumorigenesis, is significantly reduced in melanoma (130). Due to lack of MTAP expression, the processing of the metabolite MTA is impaired and thus accumulates in and outside the cell, and MTA has been described as a potential inhibitor of PRMT activity. Therefore, in melanoma cells, the loss of MTAP expression leads to a significant decrease in protein methylation through accumulation of MTA. This suggests that metabolism changes may lead to global cellular changes that support tumor development and aggressiveness (128).

## PRMT6 Inhibitors

As PRMT6 has been found to have the ability to regulate cell cycle and inhibit the expression of tumor suppressor genes in a variety of tumors, with oncogene-like properties, it is often found to be significantly overexpressed in tumor tissues, and is associated with poor prognosis. Therefore, PRMT6 can be regarded as a new research target for tumor therapy, and the study of PRMT6 inhibitors has also become a hot spot to explore potential cancer treatment approaches (**Table 1**).

**Table 1:** PRMT6 inhibitors.

Inhibitor	Structure	Mode of action	PRMT6 IC50 (nM)	Cancer type(s) tested	References
MS023	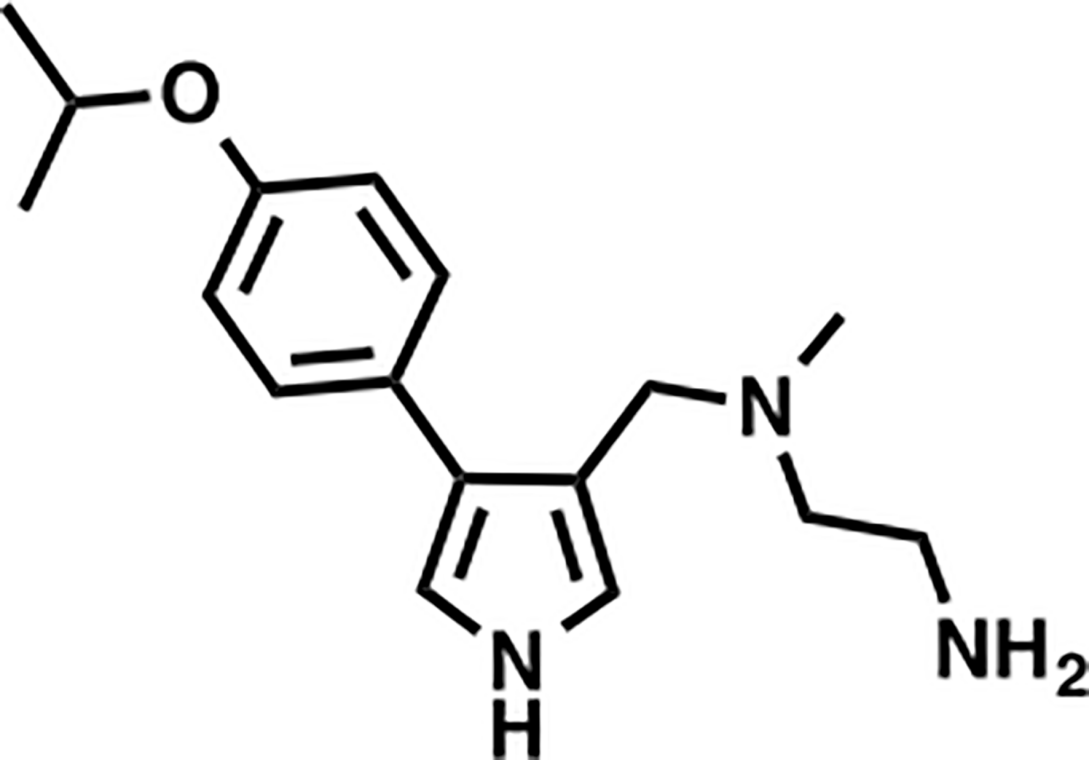	Inhibition (methyltransferase activity)	4-119	Breast cancer, colorectal cancer	(131)
EPZ020411	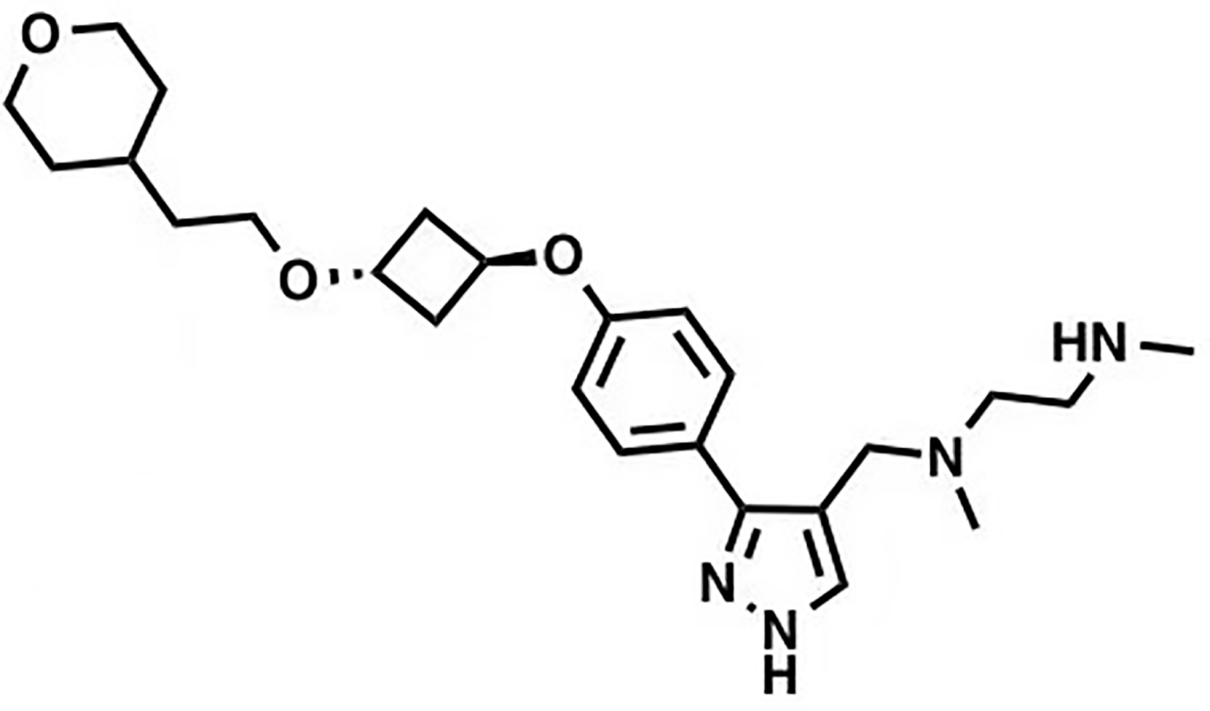	Changes of biochemical structure	10	Melanoma	(132, 133)
MS049	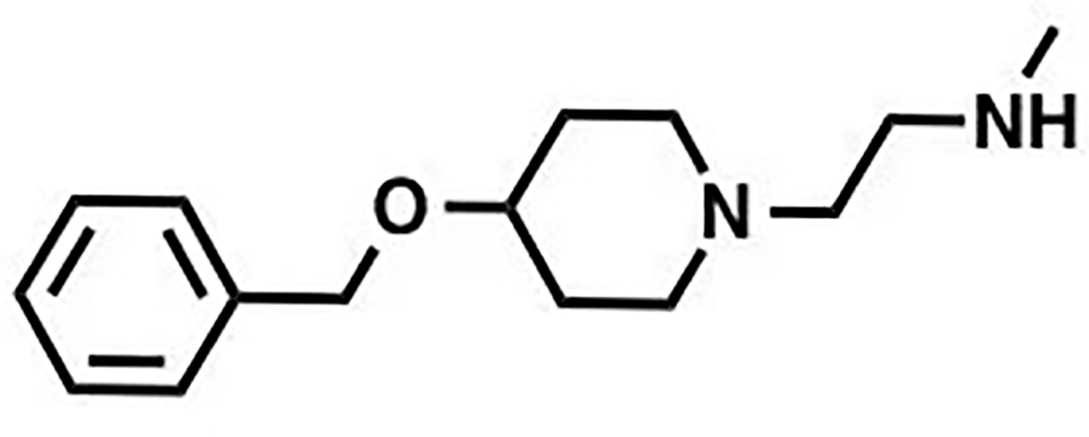	Inhibition (methyltransferase activity)	34-43	NA	(131, 133, 134)
SGC6870	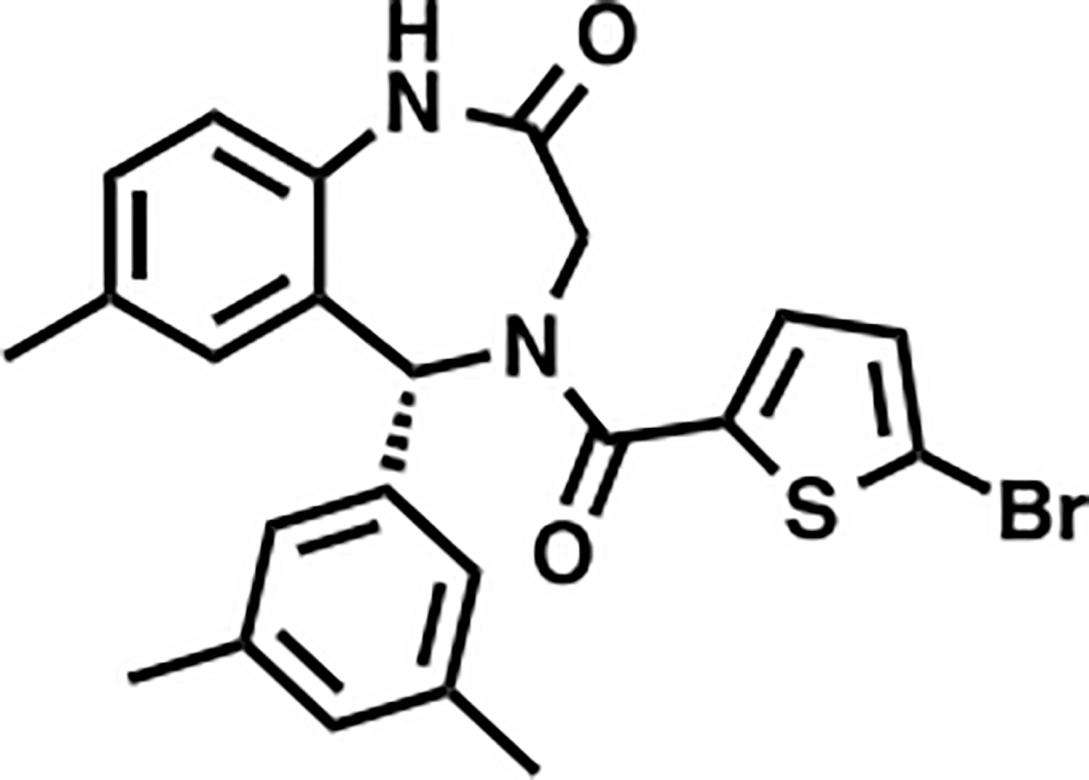	Allosteric inhibitor	77 ± 6	NA	(135)
Licochalcone A	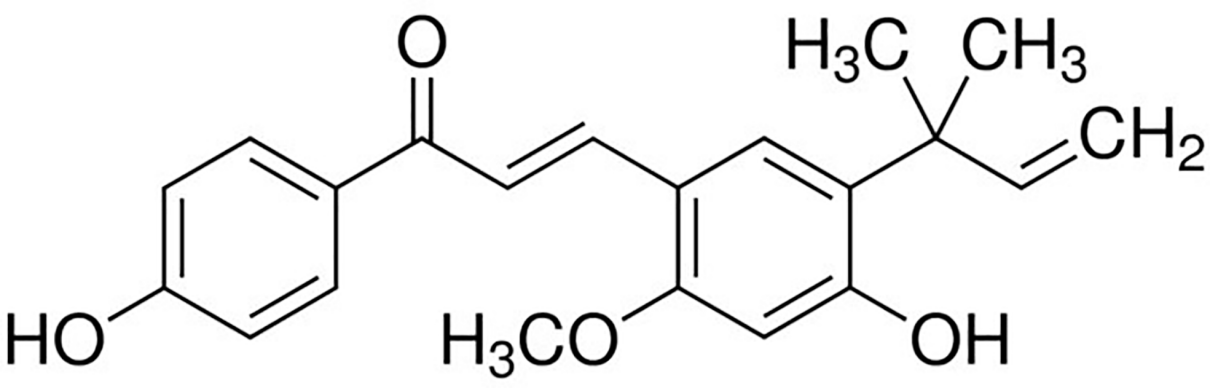	Competitive (substrates)	22.3	Breast cancer	(136)
GMS	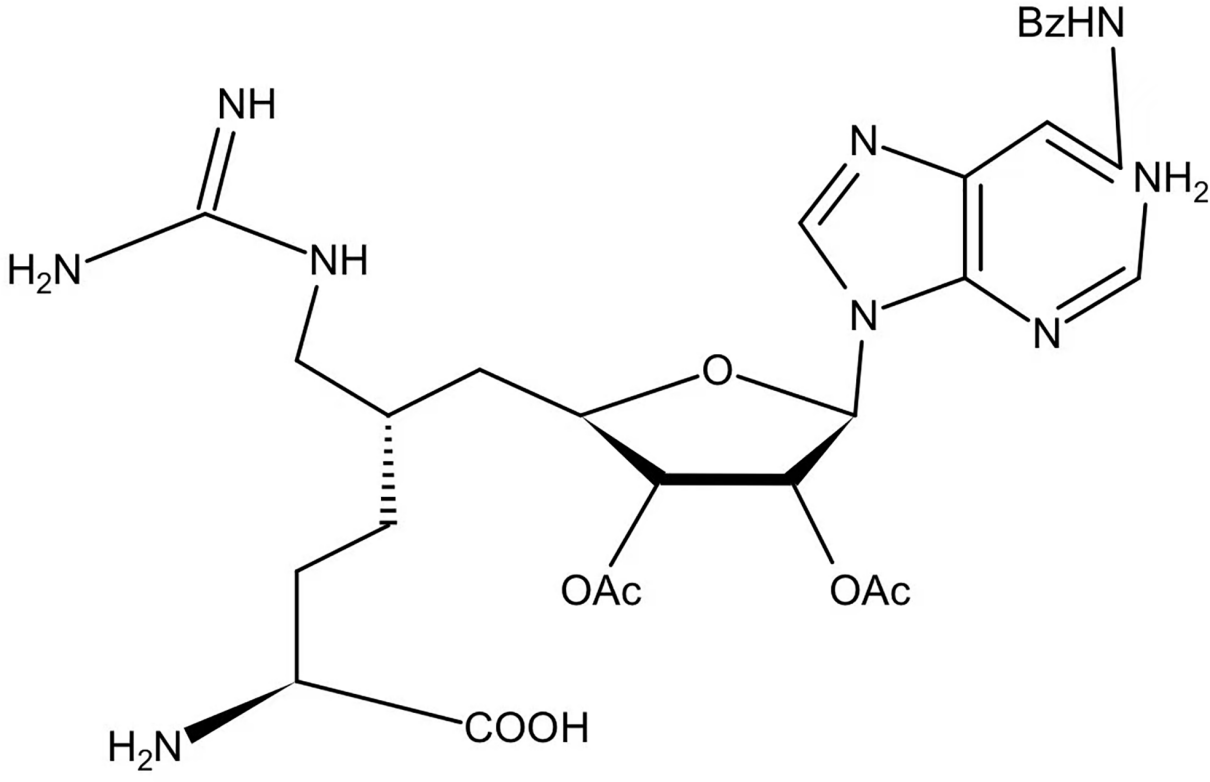	Competitive (substrates and SAM)	90	NA	(137)

NA, not available.

**Figure 2:**
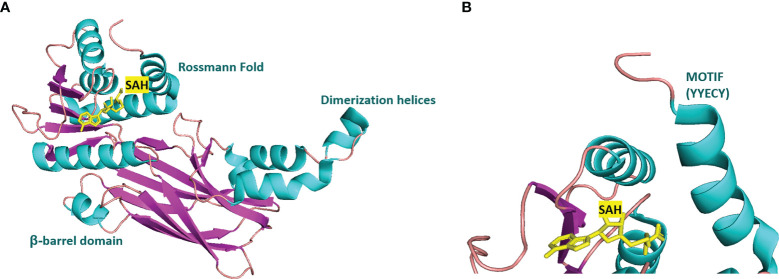
Structural attributes of PRMT6. **(A)** Overall crystal structure of human PRMT6 (PDB: 6W6D, this work) in complex with SAH (marine yellow). **(B)** Structual superimposition of the conserved motif from PRMT6 (Motif: YYECY).

Many inhibitors of PRMT6 exert their effects based on the specific structural basis of PRMT6. All the PRMT proteins have a core region of conservative catalytic domain with variable N-terminal fragment that has been observed to regulate methyl-transfer activity and substrate specificity. The PRMT6 structure consists of three structural components: (i) the N-terminal Rossmann fold, containing the SAM binding pocket, (ii) the C-terminal β-barrel domain and (iii) a dimerization helix located between the β6 and β7 strands of the C-terminal β-barrel domain, cis-conformation of cis-proline (Pro186) connects the N-terminal Rossmann fold to the C-terminal β-barrel domain (137, 138). PRMT6 exists as a dimer, with the dimerization arm (helix α4-6) from one monomer packing against helixes αY/Z and α1/2 of the Rossmann domain from the other monomers, forming a circulate dimer structure. The SAH molecule binds in an extended conformation in a pocket formed by the Rossmann fold domain. The invariant residues in Rossmann’s folded SAM binding pocket interact with the homocysteine carboxylate, adenine ring and ribose of SAH *via* a series of hydrogen bonds and salt bridges (**Figure 2**) (137). The N-terminal fragment of PRMT6 has long been thought to play an important role in substrate specificity and to be necessary and sufficient for its binding with other binding partners (139).

Several PRMT6 inhibitors have been introduced in several studies. Compounds 1-6 were found when evaluated in biochemical analysis using tritiated SAM (3H-SAM) as the methyl donor. Compound 1 with a triazole core is weak against most type I PRMTs, but it is most effective against PRMT6 (IC50 = 230 ± 12 nM). Considering that the pyrrole core is superior to triazole core in inhibiting type I PRMTs, compound 2 was found to be about 10 times more potent against PRMT6 than compound 1, and showed a higher inhibitory effect against PRMT6 (IC50 = 9 ± 0.9 nM). In addition, it was found that changing the meta-trifluoromethyl group (compound 2) to the para-isopropoxy (compound 3) improved the efficacy against all type I PRMT family. For example, MS023 (compound 3) effectively inhibited PRMT6 (IC50 = 4 ± 0.5 nM) (131). Based on the discovery of cocrystal structure of PRMT6-MS023, the first effective, selective and cytoactive irreversible inhibitor of PRMT6, compound 4 (MS117) was also reported. Mass of spectrometry (MS), kinetics studies and a eutectic structure were used to detect the covalent binding mode of compound 4 to PRMT6. Compound 4 does not covalently modify other type I PRMTs, only effectively inhibits PRMT6 in cells, and has higher selectivity to PRMT6 than other methyltransferases, and is more effective than compound 5, which is a similar analogue of compound 4 and also acts as a potent and cell-active reversible inhibitor of PRMT6 (140). At the same time, Jin et al. (140) also developed two control compounds with similar structures, 5 (MS167), a potent and cellular activity reversible PRMT6 inhibitor, and 7 (MS168), a very poor inhibitor of PRMT6, but with the same reaction warhead as compound 4.

In addition, a dual inhibitor of both CARM1 and PRMT6 called 17(MS049) was developed through structure-activity relationship (SAR) studies based on a previously designed potent selective inhibitor of the type I PRMT family inhibitor 3(MS023). The inhibition mechanism of MS049 may be as follows: (i) because MS049 has similar chemical structure to CARM1, it may occupy the substrate binding site of CARM1 and PRMT6; (ii) The binding of MS049 to the protein will cause conformational changes in the protein, which will disable the traditional enzyme kinetics (131, 134).

Based on studies of type I inhibitors, structural optimization was performed to obtain inhibitors, such as EPZ020411 with high valence (IC50 = 10 nM) and moderate selectivity (approx. 12-fold) against PRMT6, which can be used in animal models to reduce H3R2me2a levels, and the eutectic structure of PRMT6-SAH-EPZ020411 indicates that the inhibitor occupies the arginine binding site. However, as the first effective and selective small molecule tool compound of PRMT6 inhibitor, EPZ020411 is still in the stage of preclinical development, and its specific disease indications need to be further explored (132).

At the same time, systematic studies using human breast cancer cell lines demonstrated that licochalcone A, a natural compound, is a novel, reversible and selective, non-S-adenosyl L-methionine (SAM) binding site competitive PRMT6 inhibitor. In breast cancer MCF-7 cells, licochalcone A inhibits PRMT6-dependent methylation of histone H3 at arginine 2 (H3R2), resulting in a significant inhibition of estrogen receptor activity. Licochalcone A showed cytotoxicity to human MCF-7 breast cancer cells but no cytotoxicity to MCF-10A breast epithelial cells by up-regulating p53 expression, blocking G2/M cell cycle progression, and then inhibiting apoptosis. These results also indicate that licochalcone A, one of the main flavonoids extracted from licorice root, is the first natural inhibitor of PRMT6 with higher specificity (136). There is also a study identified a bisubstrate inhibitor called 6’-methyleneamine sinefungin (GMS), which is an analog of sinefungin, and its inhibitory activity is stronger than other cofactor competitive inhibitors. The compound can occupy the substrate arginine binding site (PDB:4QQK) and cofactor binding pockets (137).

It has also been reported that (R)-2 (SGC6870) was the most effective PRMT6 inhibitor (IC50 = 77 ± 6nM) in a series of more than 60 derivatives. In view of the structural differences between SGC6870 and other known substrate-competitive and SAM-competitive PRMT inhibitors. Enzyme kinetics and X-ray crystallography method validation study results show that there is a noncompetitive inhibition in the case of both the peptide substrate and SAM cofactor, suggests that it is not a SAM competitive inhibitor. SGC6870 combined without the substrate binding pocket, and the conformation of the most significant changes associated with the double-E loop, which reshaped and moved away from β-barrels of domain structure, thus forming the allosteric site. In conclusion, SGC6870 engages PRMT6 and effectively inhibits its methyltransferase activity in cells and acts as a novel allosteric inhibitor of PRMT6 (135).

## Conclusions and Perspectives

As a member of type I PRMT family, PRMT6 can regulate gene expression, promote proliferation and migration of cancer cells, activate or inhibit signal transduction, regulate cancer cell metabolism, and promote self-renewal and differentiation of tumor stem cells by methylating histone or non-histone proteins. The various biological processes associated with these functions of PRMT6 are consistent with the various proteins methylated by PRMT6 in cells. No doubt, during the course of future research, this aspect of research will be further expanded. As mentioned above, the context-specific function of PRMT6 may result from its interacting proteins. Several studies have begun to explore the binding partner proteins of PRMT6 using techniques such as co-immunoprecipitation or adjacent biotinylation, and such mechanistic studies are necessary to elucidate the importance of PRMT6-driven methylation. However, more experiments are needed to verify and explore the mechanism behind why PRMT6 overexpression has different effects on different tumor types. Therefore, further studies on PRMT6 expression levels, mechanisms of action, substrates and interaction partners, as well as interactions with other PRMT family members, will contribute to be conducted for further understanding of the function of PRMT6. At present, immunotherapy of tumor is a new hotspot with great exploration space in the field of tumor therapy. However, the research of PRMT6 in tumor immunotherapy is still weak, which provides a new research direction for cancer prevention and treatment in the future. In addition, the discoveries *in vivo* which show the specific regulation of PRMT6 is still needed to be further verified. The research of more PRMT6 substrates is also a promising study direction, which can lay more foundation for subsequent experiments. Since PRMT6 regulation occurs in the nucleus or cytoplasm, it is difficult to use PRMT6 expression as a biomarker to predict cancer status before obtaining tumor tissue. As the technical tools for drug development continue to evolve, exploratory studies on specific PRMT6 inhibitors are being improved, and the mechanisms underlying the therapeutic potential of PRMT6 inhibitors will be a promising research area. Several PRMT6 inhibitors have shown positive results in mouse models and are currently undergoing clinical trials. PRMT6 inhibitors may be used for cancer treatment alone or in combination with other current or future therapies. PRMT6 inhibitors may be beneficial in patients with tumors that have not responded to checkpoint inhibitor therapy. PRMT6 inhibitors may be very effective in cancer therapy through synergistic effects on tumor cells and tumor microenvironment components and are expected to become new tumor therapeutic targets.

**Figure 3:**
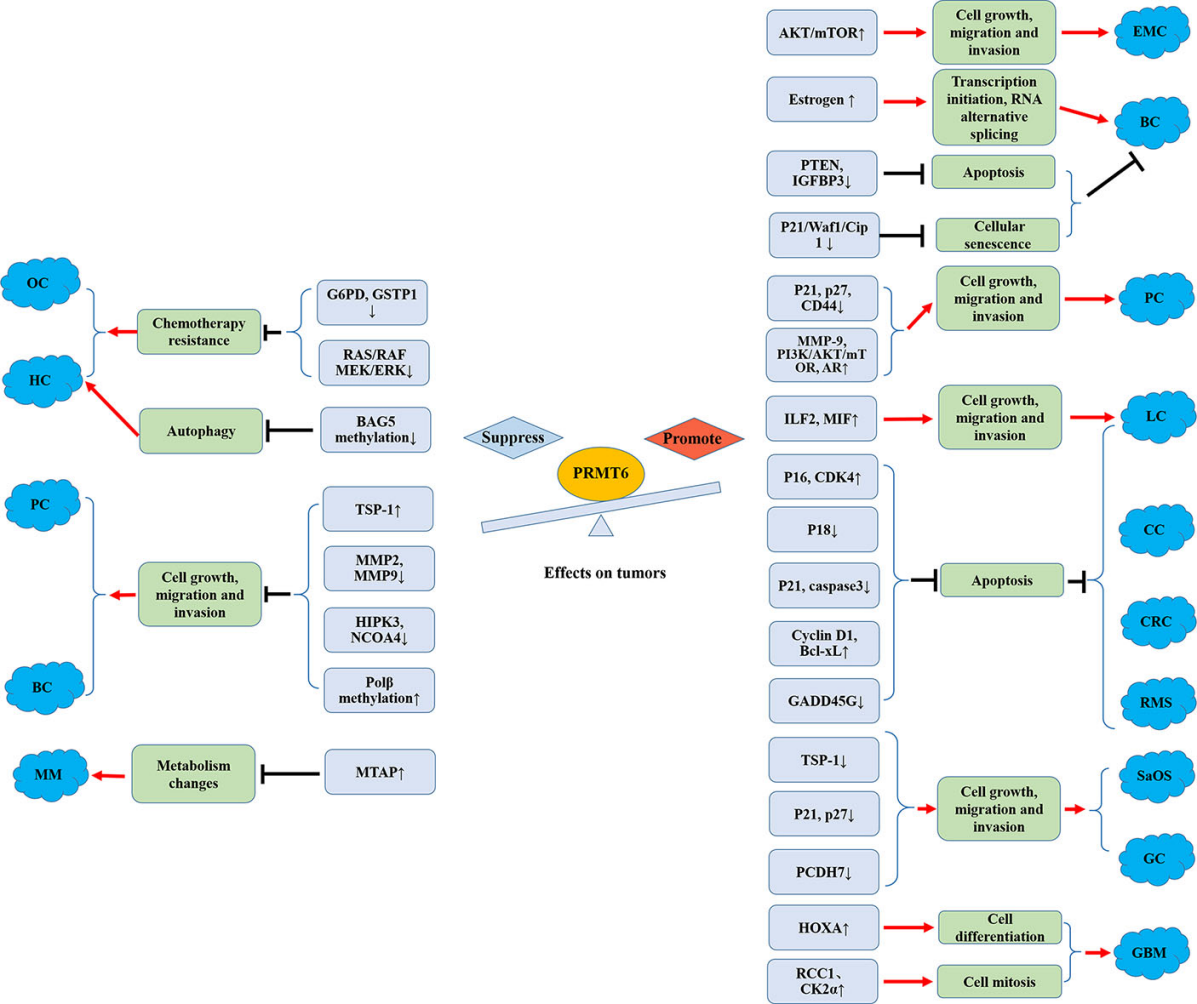
Promoting and suppressing effects of PRMT6 on different cancers. OC, ovarian cancer; HCC, hepatocellular carcinoma; MM, melanoma; PC, prostate cancer; BC, breast cancer; EMC, endometrial carcinoma; CRC, colorectal cancer; SaOS, osteosarcoma; GC, gastric cancer; CC, cervical cancer; LC, lung cancer; RMS, rhabdomyosarcoma; GBM, glioblastoma; AR, androgen receptor; Polβ, DNA polymerase β.

In conclusion, systematic approaches to arginine methylation, including the issues mentioned above (**Figure 3**), will not only help us better understand the tumor-related biological phenomena, but also help develop a novel class of anticancer drugs.

## Author Contributions

ZC and JG contributed equally to this paper. ZC: extensive literature search and drafting. JG: figures. ZW: extensive literature search. MZ and YD: literature search and critical revision. HZ: conception of the work and final version approval. All authors read and approved the final manuscript.

## Funding

This work was funded by the National Natural Science Foundation of China (grant number: 81971394, 81571457). The Shanghai Pujiang Program (grant number: 15PJ1400900).

## Conflict of Interest

The authors declare that the research was conducted in the absence of any commercial or financial relationships that could be construed as a potential conflict of interest.

## Publisher’s Note

All claims expressed in this article are solely those of the authors and do not necessarily represent those of their affiliated organizations, or those of the publisher, the editors and the reviewers. Any product that may be evaluated in this article, or claim that may be made by its manufacturer, is not guaranteed or endorsed by the publisher.
